# Relationship between orthostatic hypotension, internal carotid artery blood flow, and cognition in patients with cervical spinal cord injury

**DOI:** 10.3389/fneur.2026.1915120

**Published:** 2026-09-14

**Authors:** Jong-Joon Lee, Min-Cheol Joo

**Affiliations:** 1 Department of Rehabilitation Medicine, Wonkwang University School of Medicine, Iksan, Republic of Korea; 2 Department of Rehabilitation Medicine, Institute of Wonkwang Medical Science, Wonkwang University School of Medicine, Iksan, Republic of Korea

**Keywords:** blood circulation, cognitive dysfunction, internal carotid artery, orthostatic hypotension, spinal cord injuries

## Abstract

**Introduction:**

This study aimed to identify the distribution of orthostatic hypotension (OH) according to changes in internal carotid artery (ICA) blood flow in patients with cervical spinal cord injury (SCI), and to determine whether cognitive impairment is more prominent among patients with a certain level of ICA blood flow reduction.

**Methods:**

Forty-one patients diagnosed with cervical SCI were retrospectively analyzed and classified into two groups based on the results of a head-up tilt test and carotid duplex ultrasonography to measure ICA blood flow: those with a reduction of 21% or more (*n* = 26) and those without such a reduction (*n* = 15). Cognitive function tests were compared between the two groups.

**Results:**

Patients with an ICA blood flow reduction of 21% or greater demonstrated significantly lower cognitive scores than those without such a reduction (K-MMSE *p* = 0.028, MoCA-K *p* = 0.048; Hedges’ *g* = −0.65 for both). In multivariable regression adjusted for age, sex, and education, greater ICA blood flow reduction remained associated with lower scores on both instruments (*p* = 0.038 and *p* = 0.033). Among 13 cognitive subdomains, only language and visuospatial construction remained significantly lower after correction for multiple comparisons.

**Discussion:**

This study suggests that the early assessment of ICA blood flow and cognitive function may be informative in the rehabilitation process for patients with cervical SCI. Whether such assessment can help identify patients who should be prioritized for formal cognitive assessment requires prospective evaluation.

## 1 Introduction

Cervical spinal cord injury (SCI) is known to cause autonomic nervous system dysfunction, and orthostatic hypotension is a common complication in patients with cervical SCI (1). Orthostatic hypotension describes a drop in blood pressure that occurs with sudden changes in posture, such as standing up abruptly from a lying/sitting position, and is defined as decreases of ≥20 mmHg or ≥10 mmHg in systolic or diastolic blood pressure, respectively, within 3 min of standing, compared to the resting systolic/diastolic blood pressure in the supine position (2). It has been reported that patients with SCI with autonomic nervous system dysfunction have a decreased ability to regulate blood pressure in response to external stimuli, such as postural changes, and thus have a relatively higher probability of developing orthostatic hypotension than the general population. The prevalence of orthostatic hypotension in patients with SCI during the acute rehabilitation phase is approximately 20% in the upper thoracic cord and 74% in the cervical cord, where it is more common and tends to be more severe (3, 4).

In addition, lower cognitive performance in patients with SCI has been reported in several studies. In particular, reduced attention and executive function have been reported to be more common in cervical or upper thoracic SCIs (5), indicating that in patients with SCI, blood pressure variability due to autonomic dysfunction and cerebral perfusion instability due to impaired cerebral autoregulation may contribute to lower cognitive performance. In fact, associations of cerebral blood flow reduction upon postural change and hypotension with lower cognitive performance have been reported in patients with SCI (5). However, the number of studies and findings on this tendency remain limited, making generalization difficult, and studies in Korea are also scarce. When existing studies evaluated the association with cognition, they primarily used the degree of blood pressure change or transcranial Doppler to indirectly estimate the changes in cerebral perfusion (6, 7). The present study is distinguished from previous studies in that it directly quantified the change in internal carotid artery (ICA) blood volume flow during head-up tilt using carotid duplex ultrasonography, and analyzed the association with cognitive function by calculating the degree of change in ICA blood flow (ΔICA, %). The ICA is a major vessel that supplies approximately 70% of the total cerebral blood flow, and changes in its blood flow may reflect the state of cerebral perfusion (8).

Therefore, the present study aimed to identify the distribution of orthostatic hypotension occurrence according to ICA blood flow in patients with cervical SCI, and to examine whether lower cognitive performance appears more prominently in patients with a reduction in ICA blood flow above a certain level. A recent study reported that a reduction in ICA blood flow of approximately 21% or more after head-up tilt is significantly associated with orthostatic hypotension symptoms (9). Accordingly, we set this value as the clinical criterion for the reduction in ICA blood flow. In addition, the present study analyzed the effects of orthostatic hypotension on cognitive function. Through these analyses, we aimed to suggest the necessity of cognitive function management through ICA blood flow measurement and early intervention for orthostatic hypotension in the early rehabilitation of patients with cervical SCI.

## 2 Materials and methods

### 2.1 Subjects

This was a retrospective study of patients who received inpatient treatment for cervical SCI at the Department of Rehabilitation Medicine of Wonkwang University Hospital between November 2021 and October 2023. The inclusion criteria were adults aged 19 years or older who were diagnosed with cervical SCI radiologically or clinically and who were neurologically stable, and who underwent head-up tilt testing, cognitive function assessment, and carotid duplex ultrasonography during admission. The exclusion criteria were: (1) accompanying traumatic brain injury, (2) preexisting cognitive function impairment, and (3) active pneumonia or urinary tract infection at the time of evaluation.

### 2.2 Methods

For all subjects, demographic information (age, sex, and educational attainment) and clinical characteristics (cause, duration, and grade of SCI according to the American Spinal Injury Association classification) were extracted from the medical records. Educational attainment was converted to years of schooling and analyzed as a continuous variable (Table 1). Early in admission to the Department of Rehabilitation Medicine, to evaluate autonomic nervous system dysfunction, the presence or absence of orthostatic hypotension symptoms, systolic and diastolic blood pressure, and pulse rate were assessed through tilt table testing, and the Korean Mini-Mental State Examination and Korean Montreal Cognitive Assessment were performed (median interval, 0 days). A median of 7 days after cognitive assessment (range, −1 to 27 days), changes in ICA blood flow were measured using carotid duplex ultrasonography. Hemoglobin and hematocrit were measured a median of 2 days from ultrasonography, and medications administered on the day of cognitive assessment and on the day of ultrasonography were extracted from the electronic administration records.

**Table 1:** Baseline characteristics of the study population.

Variable	Total (*n* = 41)	Group A (*n* = 26)	Group B (*n* = 15)	*p*
Demographic and clinical characteristics				
Age (years)	60.3 ± 12.0	59.5 ± 13.6	61.7 ± 8.8	0.578^t^
Male sex	34 (82.9)	21 (80.8)	13 (86.7)	1.000ᶠ
BMI (kg/m^2^)	24.1 ± 3.1	24.8 ± 2.9	23.1 ± 3.1	0.084^t^
Education (years)	11.0 ± 4.7	10.9 ± 5.0	11.2 ± 4.3	1.000^m^
Time since injury (days)	34.8 ± 20.3	38.2 ± 20.6	28.9 ± 19.2	0.025^m^
Traumatic etiology	35 (85.4)	22 (84.6)	13 (86.7)	1.000ᶠ
Neurological level C1–C3	9 (22.0)	7 (26.9)	2 (13.3)	0.445ᶠ
AIS grade A–B	6 (14.6)	5 (19.2)	1 (6.7)	0.388ᶠ
ASIA motor score (*n* = 40)	50.7 ± 23.6	47.7 ± 23.7	55.8 ± 23.3	0.184^m^
ASIA sensory score	147.5 ± 50.2	140.4 ± 51.7	159.7 ± 46.7	0.240^t^
SCIM III	28.6 ± 21.9	24.6 ± 16.8	35.5 ± 28.1	0.210^m^
WISCI II	3.0 ± 5.5	2.4 ± 4.6	4.0 ± 6.8	0.578^m^
Hypertension	16 (39.0)	10 (38.5)	6 (40.0)	0.923^x^
Diabetes mellitus	8 (19.5)	7 (26.9)	1 (6.7)	0.220^f^
Dyslipidemia	12 (29.3)	9 (34.6)	3 (20.0)	0.480^f^
Heart disease	4 (9.8)	3 (11.5)	1 (6.7)	1.000^f^
Laboratory findings				
Hemoglobin (g/dL)	11.0 ± 1.2	11.0 ± 1.3	10.9 ± 1.1	0.745^t^
Hematocrit (%)	32.8 ± 3.8	33.3 ± 4.1	32.0 ± 3.2	0.302^t^
Medications on the day of cognitive assessment				
Anticholinergic medication	23 (56.1)	14 (53.8)	9 (60.0)	0.702^x^
Sedative medication	16 (39.0)	11 (42.3)	5 (33.3)	0.570^x^
Gabapentinoid (pregabalin or gabapentin)	27 (65.9)	16 (61.5)	11 (73.3)	0.443^x^
Total number of medications	13.0 ± 4.8	14.1 ± 4.9	11.1 ± 4.3	0.054^t^
Medications on the day of ultrasonography				
Midodrine or fludrocortisone	15 (36.6)	11 (42.3)	4 (26.7)	0.317^x^
Antihypertensive medication	10 (24.4)	6 (23.1)	4 (26.7)	1.000^f^
Orthostatic and cognitive measures				
Orthostatic hypotension	30 (73.2)	20 (76.9)	10 (66.7)	0.491^f^
Presyncopal symptoms	19 (46.3)	15 (57.7)	4 (26.7)	0.055^x^
K-MMSE	23.7 ± 4.4	22.7 ± 4.2	25.5 ± 4.3	0.028^m^
MoCA-K	20.3 ± 6.3	18.8 ± 6.3	22.9 ± 5.7	0.048^t^

Values are mean ± SD or *n* (%). Group A, ΔICA ≥21%; Group B, ΔICA <21%. ^t^Independent *t*-test; ^m^Mann–Whitney *U* test; ^f^Fisher’s exact test; ^x^Chi-square test. Fisher’s exact test was used when any expected cell count was below five. For presyncopal symptoms, Fisher’s exact test yielded *p* = 0.103. Years of education were converted as follows: no formal education, 0; elementary school, 6; middle school, 9; high school, 12; university, 16; incomplete education was assigned the last completed level. Education did not differ between groups; equivalence testing (two one-sided Welch tests) supported equivalence within ±3 years (*p* = 0.039) but not ±2 years (*p* = 0.131). Missing data: BMI (*n* = 1). The ASIA motor score could not be fully assessed in one patient owing to upper-limb splinting for a concomitant fracture and was treated as missing. Anticholinergic medication comprised antimuscarinic agents, first- and second-generation antihistamines, antipsychotics, antidepressants, and metoclopramide. Sedative medication comprised benzodiazepines, Z-drugs, opioids, centrally acting muscle relaxants, and sedating antiepileptics. Presyncopal symptoms were defined as a presyncopal symptom score of 1 or higher during head-up tilt testing.

All study procedures were conducted in accordance with the relevant standards after obtaining approval from the Institutional Review Board of Wonkwang University Hospital (IRB Number: 2024-11-005). Given the retrospective nature of the study, the requirement for informed consent was waived by the Institutional Review Board.

#### 2.2.1 Orthostatic hypotension evaluation through the tilt table test

Among the patients with cervical SCI, systolic and diastolic blood pressure and pulse rate were measured in the supine position and in the position maintained for 5 min at a 60-degree angle on a tilt table, and the presence or absence and severity of orthostatic hypotension symptoms were assessed (10). Based on these results, the patients were classified into two groups, those with and without orthostatic hypotension. Patients diagnosed with orthostatic hypotension received early management (e.g., salt supplementation, use of compression stockings, midodrine or fludrocortisone) before the ultrasonographic evaluation.

Orthostatic hypotension symptoms were assessed using the tilt table test, and the presence and severity of symptoms such as dizziness, blurred vision, and nausea were assessed. Symptoms reported by the patients during the test were documented in the medical records. Symptoms were quantified using a presyncopal symptom score, and patients reporting one or more symptoms were classified as symptomatic.

#### 2.2.2 ICA blood flow evaluation using carotid duplex ultrasonography through the tilt table test

ICA blood flow was quantitatively measured bilaterally (blood volume flow, mL/min) in patients with cervical SCI in the supine position and at 1 and 5 min at a 60-degree angle on the tilt table using a Vivid E95 device (GE Healthcare, USA), according to the standard examination guidelines for carotid duplex ultrasonography (11). All examinations were performed by a single sonographer with dedicated training in vascular ultrasound. Measurements were obtained from a straight segment of the internal carotid artery at least 2 cm distal to the carotid bulb; peak systolic velocity, end-diastolic velocity, time-averaged mean velocity (TAMEAN), and vessel diameter were each measured three times at each time point on each side, and the mean of the three measurements was used (8). Volume flow was calculated as vessel cross-sectional area (cm^2^) × TAMEAN (cm/s) × 60; ΔICA is based on volume flow, and the velocity and diameter measurements are reported in Table 2. Measurement at 5 min followed the protocol of the previous study from which the 21% threshold was derived (9), in which autoregulatory adjustment to postural change was considered to have stabilized by that time; the 1-min measurement was added to capture the early transient response.

**Table 2:** Internal carotid artery hemodynamic parameters in the supine position and during head-up tilt.

Side	Parameter	Supine	Tilt 1 min	*p*	Tilt 5 min	*p*
Right	PSV (cm/s)	68.2 ± 19.3	67.9 ± 19.1	0.885^t^	68.5 ± 19.9	0.848^t^
Right	EDV (cm/s)	26.9 ± 8.7	23.5 ± 8.0	<0.001^t^	22.5 ± 7.8	<0.001^t^
Right	TAMEAN (cm/s)	29.6 ± 9.8	25.9 ± 8.8	<0.001^t^	25.9 ± 8.1	<0.001^t^
Right	Diameter (mm)	5.09 ± 0.54	5.11 ± 0.58	0.477^w^	5.12 ± 0.57	0.509^w^
Right	Volume flow (mL/min)	352.7 ± 138.0	319.7 ± 129.6	0.004^t^	317.2 ± 124.2	0.002^t^
Left	PSV (cm/s)	68.4 ± 16.7	70.2 ± 20.1	0.309^t^	70.4 ± 20.8	0.244^t^
Left	EDV (cm/s)	26.5 ± 6.3	24.1 ± 6.8	<0.001^w^	23.6 ± 7.5	<0.001^t^
Left	TAMEAN (cm/s)	28.6 ± 7.0	26.4 ± 7.0	0.002^w^	26.7 ± 8.2	0.010^w^
Left	Diameter (mm)	5.16 ± 0.62	5.14 ± 0.57	0.897^w^	5.17 ± 0.56	0.363^w^
Left	Volume flow (mL/min)	363.4 ± 79.2	322.7 ± 85.5	<0.001^t^	331.6 ± 94.0	0.008^t^
Right	ΔICA (%)	–	7.3 ± 21.0	–	8.1 ± 20.4	–
Left	ΔICA (%)	–	10.6 ± 16.2	–	8.0 ± 19.0	–

Values are mean ± SD (*n* = 41). *p* values compare each tilt time point with the supine measurement. ^t^Paired *t*-test; ^w^Wilcoxon signed-rank test, used when the distribution of differences deviated from normality. PSV, peak systolic velocity; EDV, end-diastolic velocity; TAMEAN, time-averaged mean velocity. ΔICA denotes the percentage reduction in volume flow from the supine value at each time point, calculated for each patient; negative values indicate an increase, and no supine-versus-tilt comparison applies. The maximum of the four ΔICA values, used for group classification, was 23.4 ± 15.2% overall: 32.4 ± 9.7% in Group A and 7.8 ± 8.9% in Group B.

The degree of change in ICA blood flow (ΔICA) was calculated using the following formula: [(supine blood flow − tilt blood flow)/supine blood flow] × 100 (%). The ΔICA was calculated separately for the bilateral ICAs at 1 and 5 min, and patients in whom any of these four values decreased by 21% or more were classified as Group A, while patients in whom all four values decreased by less than 21% were classified as Group B. The threshold was adopted from a previous study (9), which derived it from the 5-min measurement only; the present study used the maximum across both sides and both time points to capture side-to-side and time-point variability in the orthostatic response. Sensitivity analyses using alternative definitions of ΔICA are presented in Supplementary Table S2. Carotid plaque and stenosis were assessed as part of the routine duplex examination but were not analyzed in the present study.

#### 2.2.3 Cognitive function evaluation using K-MMSE and MoCA-K

The Korean Mini-Mental State Examination (K-MMSE) and the Korean Montreal Cognitive Assessment (MoCA-K) were used to evaluate cognitive function. The maximum score for each test was 30 points. The K-MMSE comprises 6 subdomains: orientation to time (5 points), orientation to place (5 points), registration (3 points), attention and calculation (5 points), recall (3 points), and language and visuospatial construction (9 points). The MoCA-K comprises 6 subdomains: visuospatial/executive function (5 points), naming (3 points), attention (6 points), language (3 points), abstraction and delayed recall (7 points), and orientation (6 points). In this study, we compared and analyzed the total scores and the scores of each subdomain. Among the subdomains of the MoCA-K, abstraction and delayed recall (7 points) was divided into abstraction (2 points) and delayed recall (5 points) for analysis.

#### 2.2.4 Statistical analysis

Statistical analysis was performed using the SPSS statistical program ver. 27.0 (IBM SPSS, Armonk, NY, USA) and Python 3.12 (scipy 1.14, statsmodels 0.14), and the level of statistical significance was set at *p* < 0.05 (two-tailed).

For the comparison of demographic and clinical characteristics between the two groups, the independent samples *t*-test was used for continuous variables when normality (Shapiro–Wilk test) and equality of variances (Levene test) were satisfied; Welch’s *t*-test was used when variances were unequal, and the Mann–Whitney *U* test when normality was violated. The test used for each variable is indicated in the tables. For categorical variables, the chi-square test was used, or Fisher’s exact test when any expected cell count was below five. The proportion of patients who showed orthostatic hypotension symptoms and those who were actually diagnosed in each group were analyzed in the same manner. The comparison of the total and subdomain scores of the K-MMSE and the MoCA-K between the groups was analyzed using the same rule; Hedges’ *g* with 95% confidence intervals is reported for all group comparisons, and the 13 subdomain *p* values were corrected for multiple comparisons using the Benjamini–Hochberg false discovery rate procedure.

Medication exposure was recorded as binary variables in the six categories shown in Table 1, together with the total number of prescribed medications. For medications that could directly affect cognitive testing (cholinesterase inhibitors or choline alfoscerate), the timing and indication of each prescription were verified against the administration records and with the treating physicians.

The unadjusted association between ΔICA, analyzed as a continuous variable, and each cognitive score was assessed with the Pearson correlation coefficient; Spearman coefficients were also computed to confirm that the results were not driven by distributional assumptions. In addition, multivariable linear regression analysis was conducted with ΔICA as a continuous variable, expressed per 10-percentage-point reduction, to adjust for confounding variables, including age, sex, and years of education, specified in advance. Other candidate variables—time since injury, orthostatic hypotension, hemoglobin, injury completeness, and the medication variables—were examined one at a time in sensitivity models and are reported in the Supplementary material. To examine whether ΔICA and presyncopal symptoms carry independent information, both were entered together in the adjusted model as an exploratory analysis. Bootstrap confidence intervals (5,000 resamples, percentile method) are reported alongside conventional intervals because the residuals of the MoCA-K model deviated from normality; model diagnostics (variance inflation factors, Breusch–Pagan test, Cook’s distance) and leave-one-out analyses were also performed (Supplementary Table S3).

The discriminative performance of ΔICA was evaluated using receiver operating characteristic (ROC) analysis against four outcomes: K-MMSE < 24, MoCA-K < 23, the presence of presyncopal symptoms, and the diagnosis of orthostatic hypotension; areas under the curve are reported with bootstrap 95% confidence intervals, and optimal thresholds were identified using the Youden index. The dependence of the findings on the 21% threshold was examined across cutoffs from 10 to 35%.

## 3 Results

### 3.1 General characteristics of the study subjects

The 41 patients (Figure 1) included 34 males and 7 females, with an age distribution ranging from 24 to 83 years, and a mean age of 60.3 ± 12.0 years. The distribution by cause of SCI was 6 patients with disease and 35 patients with trauma, and the duration from SCI to evaluation ranged from 10 to 109 days, with a mean of 34.8 ± 20.3. The distribution of the SCI site was 1, 3, 5, 21, and 11 patients for cervical levels 1 through 5, respectively, and the distribution by functional level of the injured site according to the American Spinal Injury Association Impairment Scale (AIS) classification was 3 patients each in the complete motor paralysis group grades A and B, and 13 and 22 patients in the incomplete motor paralysis group grades C and D, respectively (Table 1). Educational attainment averaged 11.0 ± 4.7 years (Table 1). Baseline characteristics, including the six medication variables and the total number of prescribed medications, did not differ significantly between Groups A and B, except that the duration from injury was longer in Group A (38.2 ± 20.6 versus 28.9 ± 19.2 days; *p* = 0.025 by the Mann–Whitney *U* test, *p* = 0.162 by the *t*-test; Table 1).

**Figure 1:**
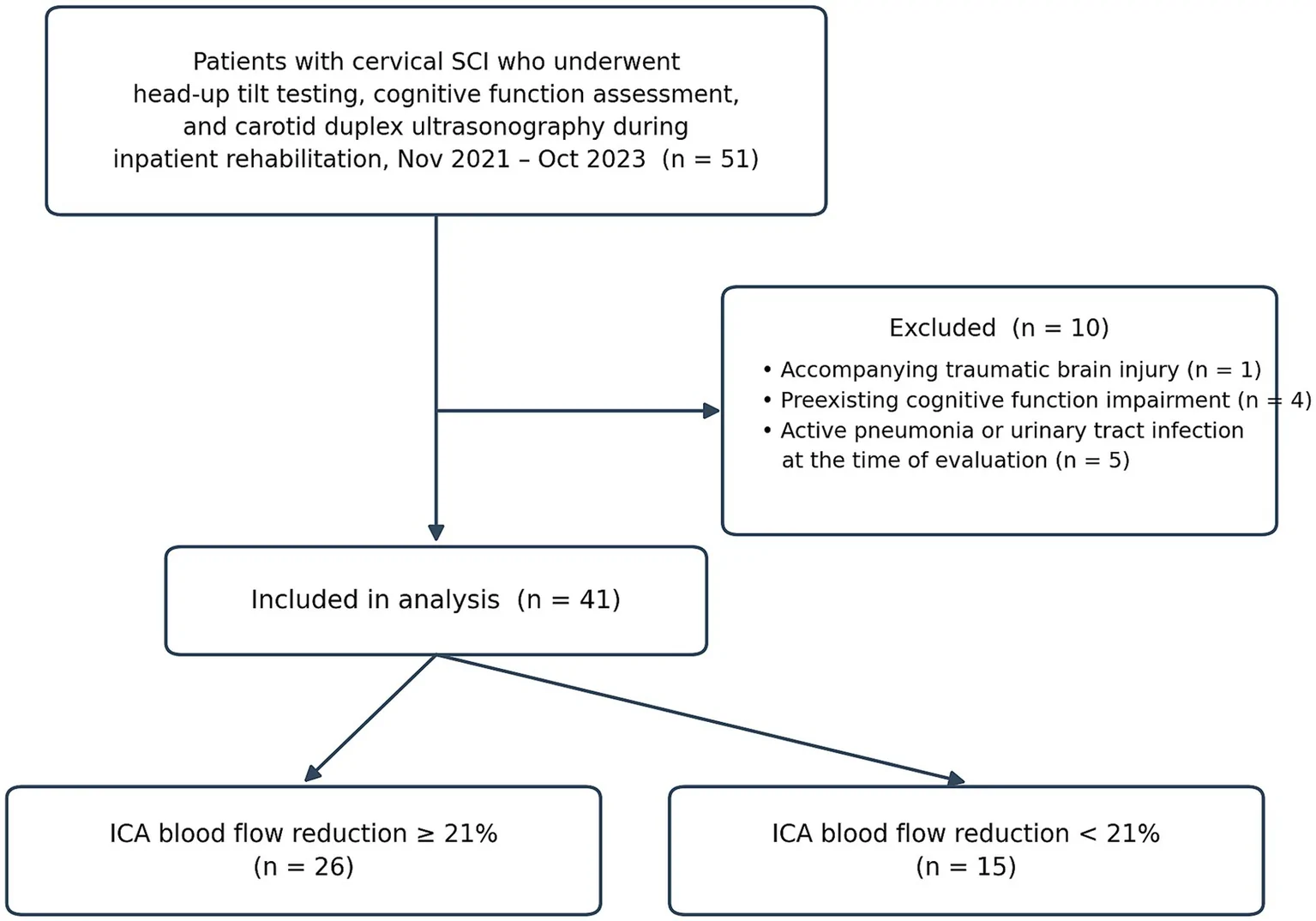
Flowchart of patient selection. Of 51 patients with cervical spinal cord injury (SCI) who underwent head-up tilt testing, cognitive function assessment, and carotid duplex ultrasonography during inpatient rehabilitation between November 2021 and October 2023, 10 were excluded, leaving 41 patients for analysis. Patients were classified according to the reduction in internal carotid artery (ICA) blood flow during head-up tilt (≥21%, *n* = 26; <21%, *n* = 15).

### 3.2 ICA hemodynamic parameters during the head-up tilt test

Compared with the supine position, peak systolic velocity and vessel diameter did not change significantly at 1 or 5 min of head-up tilt on either side, whereas end-diastolic velocity and TAMEAN decreased significantly and bilaterally at both time points (all *p* ≤ 0.010). Volume flow decreased accordingly on both sides (Table 2). Representative Doppler waveforms in the supine position and during head-up tilt are shown in Figure 2.

**Figure 2:**
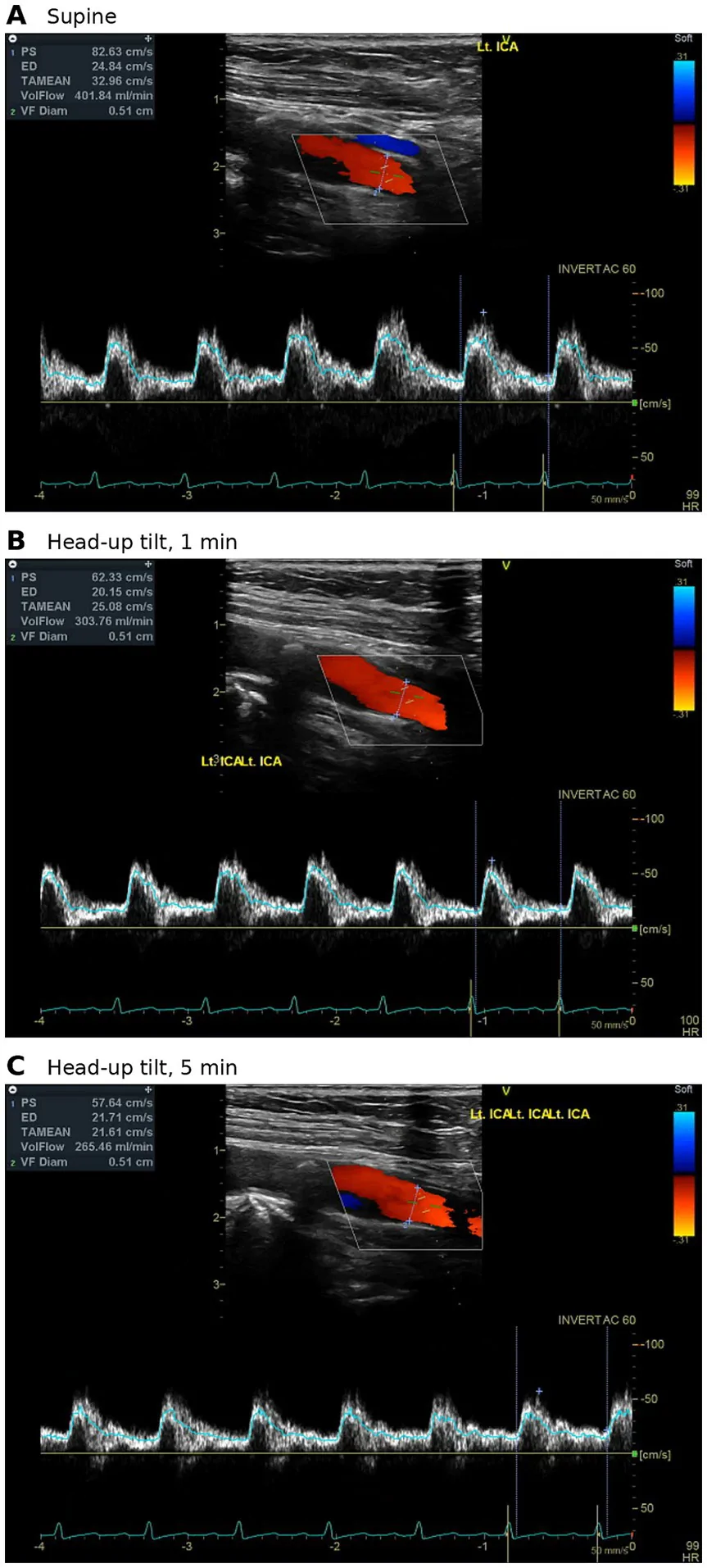
Representative Doppler waveforms of the internal carotid artery (ICA). Spectral Doppler tracings of the left ICA from a representative patient in the ΔICA ≥21% group, obtained in the supine position **(A)** and at 1 min **(B)** and 5 min **(C)** of 60° head-up tilt. Peak systolic velocity (PS), end-diastolic velocity (ED), time-averaged mean velocity (TAMEAN), volume flow, and vessel diameter are shown on each display. TAMEAN and volume flow decreased progressively during head-up tilt (32.96 → 25.08 → 21.61 cm/s and 401.84 → 303.76 → 265.46 mL/min, respectively), whereas vessel diameter was unchanged (0.51 cm).

### 3.3 Association between change in carotid blood flow volume and orthostatic hypotension

Among the 26 patients in Group A, 20 (76.9%) were diagnosed with orthostatic hypotension through blood pressure measurement, and 15 (57.7%) reported presyncopal symptoms. Among the 15 patients in Group B, 10 (66.7%) were diagnosed with orthostatic hypotension through blood pressure measurement, and 4 (26.7%) reported symptoms. The diagnosis rate (*p* = 0.491) and symptom occurrence rate (*p* = 0.055) of orthostatic hypotension were not significantly different between the two groups (Table 3).

**Table 3:** Orthostatic hypotension and presyncopal symptoms according to ΔICA group.

Variable	Status	Group A (*n* = 26)	Group B (*n* = 15)	*p*
Orthostatic hypotension	(+)	20 (76.9)	10 (66.7)	0.491
	(−)	6 (23.1)	5 (33.3)	
Presyncopal symptoms	(+)	15 (57.7)	4 (26.7)	0.055
	(−)	11 (42.3)	11 (73.3)	

Values are *n* (%). Group A, ΔICA ≥21%; Group B, ΔICA <21%. The chi-square test was used for presyncopal symptoms (all expected cell counts ≥5) and Fisher’s exact test for orthostatic hypotension (one expected cell count <5); Fisher’s exact test for presyncopal symptoms yielded *p* = 0.103.

### 3.4 Association between the change in carotid blood flow volume and cognitive function

The cognitive function evaluation results of all subjects were 23.73 ± 4.43 points on the K-MMSE and 20.32 ± 6.32 points on the MoCA-K. When divided into Group A and Group B for comparison, the K-MMSE score was 22.69 ± 4.24 points in Group A and 25.53 ± 4.31 points in Group B, with Group A significantly lower (*p* = 0.028; Hedges’ *g* = −0.65). The corresponding MoCA-K scores were 18.85 ± 6.28 points and 22.87 ± 5.71, respectively, with Group A significantly lower (*p* = 0.048; Hedges’ *g* = −0.65; Table 4, Figure 3).

**Table 4:** Cognitive function according to ΔICA group.

Test	Group A (*n* = 26)	Group B (*n* = 15)	Mean difference (95% CI)	*p*	Hedges’ *g* (95% CI)
K-MMSE	22.69 ± 4.24	25.53 ± 4.31	−2.84 (−5.64, −0.04)	0.028^m^	−0.65 (−1.29, −0.01)
MoCA-K	18.85 ± 6.28	22.87 ± 5.71	−4.02 (−8.01, −0.03)	0.048^t^	−0.65 (−1.29, −0.01)

Values are mean ± SD. ^m^Mann–Whitney *U* test (K-MMSE: Shapiro–Wilk *p* = 0.032 in Group B); ^t^Independent *t*-test (MoCA-K: Shapiro–Wilk *p* = 0.135 and 0.247; Levene *p* = 0.641). For the MoCA-K, the Mann–Whitney *U* test yielded *p* = 0.051; parametric assumptions were satisfied and the *t*-test is reported as the primary result.

**Figure 3:**
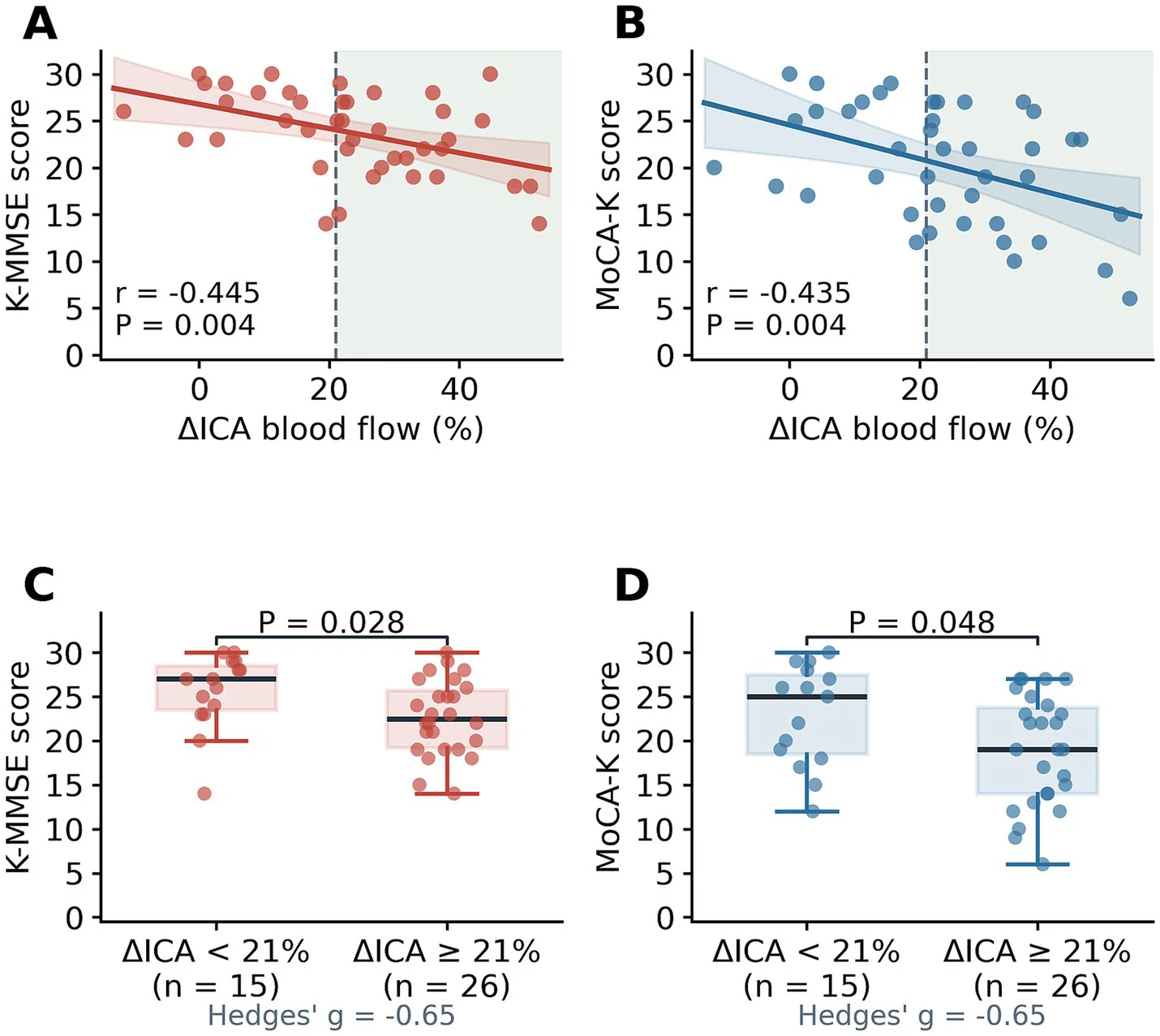
Association between the postural reduction in internal carotid artery (ICA) blood flow (ΔICA) and cognitive scores. **(A,B)** Scatter plots of ΔICA against K-MMSE **(A)** and MoCA-K **(B)** scores, with the fitted regression line and its 95% confidence band; *r* denotes the Pearson correlation coefficient. The dashed vertical line marks the 21% threshold, and the shaded area indicates the ΔICA ≥21% range. **(C,D)** K-MMSE **(C)** and MoCA-K **(D)** scores according to ΔICA group (<21%, *n* = 15; ≥21%, *n* = 26). Boxes indicate the median and interquartile range, whiskers the range of values excluding outliers, and dots individual patients. *p* values are from the Mann–Whitney *U* test **(C)** and the independent *t*-test **(D)**; Hedges’ *g* = −0.65 for both comparisons.

As a continuous variable, ΔICA was inversely correlated with both scores (K-MMSE, *r* = −0.445, *p* = 0.004; MoCA-K, *r* = −0.435, *p* = 0.004; Figure 3). In multivariate linear regression analysis adjusted for age, sex, and years of education, each 10-percentage-point greater reduction in ICA blood flow was associated with a 0.83-point lower K-MMSE score (95% CI −1.61 to −0.05; *p* = 0.038) and a 1.23-point lower MoCA-K score (95% CI −2.36 to −0.10; *p* = 0.033; Table 5). In sensitivity models adjusting individually for time since injury, orthostatic hypotension, hemoglobin, injury completeness, and the medication variables, the ΔICA coefficient ranged from −0.73 to −1.03 for the K-MMSE (all *p* ≤ 0.08; Supplementary Tables S4, S5). In leave-one-out analyses, the direction of the ΔICA coefficient was unchanged in all 41 iterations, although statistical significance was not retained in 11 of 41 iterations for the K-MMSE and 5 of 41 for the MoCA-K.

**Table 5:** Multivariable linear regression of cognitive score on ΔICA.

(a) Summary of ΔICA coefficients					
Outcome	Model	*β* (95% CI)	Bootstrap 95% CI	*p*	Adjusted *R*^2^
K-MMSE	Unadjusted	−1.30 (−2.15, −0.45)	−2.08, −0.47	0.004	0.178
	Adjusted	−0.83 (−1.61, −0.05)	−1.71, −0.15	0.038	0.381
MoCA-K	Unadjusted	−1.81 (−3.02, −0.60)	−3.17, −0.59	0.004	0.169
	Adjusted	−1.23 (−2.36, −0.10)	−2.56, −0.14	0.033	0.362

(b) Full coefficients of the adjusted models				
Covariate	K-MMSE *β* (95% CI)	*p*	MoCA-K *β* (95% CI)	*p*
ΔICA (per 10%)	−0.83 (−1.61, −0.05)	0.038	−1.23 (−2.36, −0.10)	0.033
Age (years)	0.04 (−0.08, 0.15)	0.528	−0.04 (−0.21, 0.12)	0.611
Male sex	−0.88 (−4.24, 2.48)	0.599	−0.42 (−5.29, 4.45)	0.861
Education (years)	0.56 (0.23, 0.90)	0.002	0.63 (0.15, 1.11)	0.012

(a) *β* is expressed per 10-percentage-point reduction in ICA blood flow. Adjusted models include age, sex, and years of education. Bootstrap confidence intervals were derived from 5,000 resamples (percentile method).

(b) Model diagnostics: all variance inflation factors <2.0; Breusch–Pagan (*F* statistic) *p* = 0.926 (K-MMSE) and 0.186 (MoCA-K); maximum Cook’s distance 0.359 and 0.157; residual Shapiro–Wilk *p* = 0.280 and 0.005.

Fifteen patients were receiving midodrine or fludrocortisone at the time of ultrasonography; these patients showed larger reductions in ICA blood flow (29.1% versus 20.1%, *p* = 0.065), and adjustment for their use did not attenuate the association (*β* = −0.93, *p* = 0.030). Cholinesterase inhibitors or choline alfoscerate had been prescribed in four patients, in every case initiated after the cognitive assessment or for indications unrelated to cognition (tremor; restless legs syndrome); no patient was receiving cognition-modifying medication at the time of cognitive testing. Systemic corticosteroids were administered to five patients, all in Group A, for cutaneous adverse drug reactions.

### 3.5 Cognitive subdomain analysis of K-MMSE and MoCA-K

In the subdomain analysis of the K-MMSE, Group A showed nominally lower scores than Group B in the attention and calculation (*p* = 0.042) and language and visuospatial construction domains (*p* = 0.003). In the subdomain analysis of the MoCA-K, Group A scored nominally lower than Group B in the delayed recall domain (*p* = 0.026; Table 6). After correction for multiple comparisons across the 13 subdomains, only language and visuospatial construction remained significant (adjusted *p* = 0.035; Table 6).

**Table 6:** Cognitive subdomain scores according to ΔICA group, with correction for multiple comparisons.

Domain	Group A (*n* = 26)	Group B (*n* = 15)	Hedges’ *g* (95% CI)	*p* ^t^	*p* ^m^	FDR *p*
K-MMSE subdomains						
Orientation to time (5)	3.92 ± 1.29	4.27 ± 1.10	−0.27 (−0.90, 0.35)	0.393	0.336	0.437
Orientation to place (5)	4.69 ± 0.93	4.87 ± 0.52	−0.21 (−0.84, 0.41)	0.508	0.617	0.702
Registration (3)	2.96 ± 0.20	2.87 ± 0.52	0.27 (−0.36, 0.89)	0.404	0.690	0.702
Attention and calculation (5)	2.23 ± 1.99	3.53 ± 1.81	−0.66 (−1.30, −0.02)	0.043	0.042	0.180
Recall (3)	1.58 ± 1.03	1.73 ± 0.88	−0.16 (−0.78, 0.47)	0.624	0.702	0.702
Language and visuospatial construction (9)	7.31 ± 1.52	8.27 ± 1.28	−0.65 (−1.29, −0.01)	0.046	0.003	0.035
MoCA-K subdomains						
Visuospatial/executive (5)	2.08 ± 2.00	3.00 ± 2.17	−0.44 (−1.07, 0.19)	0.175	0.123	0.348
Naming (3)	2.85 ± 0.46	3.00 ± 0.00	−0.41 (−1.04, 0.22)	0.209	0.187	0.348
Attention (6)	4.08 ± 1.76	4.87 ± 1.46	−0.47 (−1.10, 0.17)	0.151	0.171	0.348
Language (3)	2.38 ± 0.75	2.67 ± 0.49	−0.41 (−1.04, 0.22)	0.202	0.252	0.364
Abstraction (2)	1.23 ± 0.86	1.60 ± 0.74	−0.44 (−1.07, 0.19)	0.173	0.159	0.348
Delayed recall (5)	1.23 ± 1.66	2.33 ± 1.63	−0.66 (−1.30, −0.02)	0.046	0.026	0.167
Orientation (6)	5.00 ± 1.30	5.40 ± 1.06	−0.32 (−0.95, 0.30)	0.316	0.235	0.364

Values are mean ± SD; maximum attainable score in parentheses. ^t^Independent *t*-test, as in the original submission; ^m^Mann–Whitney *U* test, the primary test, applied because the Shapiro–Wilk test indicated non-normality for all 13 subdomains. Both are shown so that the effect of the choice of test is visible. FDR, Benjamini–Hochberg false discovery rate across the 13 Mann–Whitney comparisons. Only language and visuospatial construction remained significant after Benjamini–Hochberg, Holm, or Bonferroni adjustment.

### 3.6 Association between orthostatic hypotension, presyncopal symptoms, and cognitive function

Regarding the K-MMSE, the scores of the groups with and without orthostatic hypotension were 23.70 ± 3.81 and 23.82 ± 6.05 points, respectively, showing no statistically significant difference (*p* = 0.953). For the MoCA-K, the respective scores were 20.00 ± 6.21 and 21.18 ± 6.82 points, respectively, likewise showing no statistically significant difference (*p* = 0.602; Table 7).

**Table 7:** Cognitive function according to orthostatic hypotension and to presyncopal symptom status.

(a) By blood-pressure–based diagnosis of orthostatic hypotension					
Test	OH (+) (*n* = 30)	OH (−) (*n* = 11)	Mean difference (95% CI)	*p*	Hedges’ *g*
K-MMSE	23.70 ± 3.81	23.82 ± 6.05	−0.12 (−3.32, 3.08)	0.953^w^	−0.03
MoCA-K	20.00 ± 6.21	21.18 ± 6.82	−1.18 (−5.73, 3.36)	0.602^t^	−0.18

(b) By presyncopal symptom status				
Test	Symptoms (+) (*n* = 19)	Symptoms (−) (*n* = 22)	*p*	Hedges’ *g*
K-MMSE	22.21 ± 4.55	25.05 ± 3.97	0.040^t^	−0.65
MoCA-K	18.84 ± 6.41	21.59 ± 6.10	0.168^t^	−0.43

Values are mean ± SD. ^w^Welch’s *t*-test (Levene *p* = 0.035); ^t^Independent *t*-test. Presyncopal symptoms were defined as a presyncopal symptom score of 1 or higher during head-up tilt testing.

In contrast, patients reporting presyncopal symptoms scored lower on the K-MMSE than those without (22.21 ± 4.55 versus 25.05 ± 3.97; *p* = 0.040; Hedges’ *g* = −0.65), with a smaller, nonsignificant difference on the MoCA-K (18.84 ± 6.41 versus 21.59 ± 6.10; *p* = 0.168; *g* = −0.43; Table 7). In the exploratory model entering ΔICA and symptom status together, both coefficients were attenuated (variance inflation factors for the two variables below 1.3); symptom status remained statistically significant (*β* = −2.48, *p* = 0.037), whereas ΔICA did not (*β* = −0.57, *p* = 0.148).

### 3.7 Discriminative performance of ΔICA and evaluation of the 21% threshold

ΔICA discriminated K-MMSE scores below 24 with an area under the curve of 0.718 (bootstrap 95% CI 0.545–0.878) and a Youden-optimal threshold of 22.8%; at the *a priori* 21% threshold, sensitivity was 0.79 and specificity 0.50. Discrimination was not statistically significant for MoCA-K below 23 (AUC 0.627, 95% CI 0.442–0.813), presyncopal symptoms (AUC 0.656, 95% CI 0.476–0.821), or the diagnosis of orthostatic hypotension (AUC 0.609, 95% CI 0.410–0.797; Supplementary Figure S1). Among the three outcomes for which ΔICA showed nominal discrimination, Youden-optimal thresholds ranged from 18.7 to 22.8%, encompassing the 21% value adopted *a priori*; for orthostatic hypotension, the Youden-derived threshold was 32.9% with a sensitivity of 0.37. Across cutoffs from 10 to 35%, the adjusted association between the dichotomized ΔICA and the K-MMSE remained significant between 15 and 25% but not at the extremes (Supplementary Table S1).

## 4 Discussion

The prevalence of orthostatic hypotension in patients with cervical SCI is approximately 60–74% (3), making it a major complication that hinders neurological recovery and the effective progression of rehabilitation treatment (12). The risk of cognitive impairment in patients with SCI has been reported to be approximately 13 times higher than that in non-injured individuals (13), while lower cognitive performance is observed in approximately 10–64% of patients, becoming a major factor that hinders participation in rehabilitation programs and treatment effectiveness (14). Approximately 50% of patients with traumatic SCI and brain injury show reduced attention, executive function, and working memory (15–17). However, a similar pattern of lower cognitive performance has been consistently reported in patients with SCI without an accompanying brain injury (13). This suggests that cerebral perfusion instability due to autonomic nervous system dysfunction may independently contribute to lower cognitive performance.

Autonomic nervous system dysfunction in patients with SCI leads to a reduction in cerebral autoregulation, cerebrovascular reactivity to carbon dioxide, and neurovascular coupling (NVC), and can consequently induce cognitive impairment due to cerebrovascular dysfunction (6, 18). Neurovascular coupling is a physiological mechanism by which local cerebral blood flow is regulated to increase when neural activity increases. It is known that the higher the level of SCI, the more severe the degree of autonomic nervous system dysfunction. Due to increased blood pressure variability, the perfusion pressure to the brain becomes unstable, leading to a decrease in oxygen supply and metabolic activity (6, 18).

According to prior studies, patients with SCI and orthostatic hypotension showed significantly lower scores in several cognitive domains, including memory, attention, information processing speed, and executive function, than the general group of patients with SCI (7, 13, 19). In one study by Phillips et al., changes in the blood flow velocity of the middle cerebral artery (MCA) were measured non-invasively using transcranial Doppler, and neurovascular coupling (NVC) was assessed by simultaneously performing executive function tasks. They reported that, under conditions of orthostatic hypotension, the ability to perform executive function tasks was reduced along with a decrease in NVC, and after midodrine administration, NVC and executive function were significantly improved, along with the recovery of blood pressure (7). This suggests that the reduction in cerebral blood flow and metabolism due to hypotension is closely related to executive function.

In the present study, the relationship between the degree of ICA blood flow reduction and cognitive function in patients with cervical SCI was statistically significant. In multivariate linear regression analysis adjusted for age, sex, and years of education, each 10-percentage-point greater reduction in ICA blood flow was associated with a 0.83-point lower K-MMSE score (*p* = 0.038) and a 1.23-point lower MoCA-K score (*p* = 0.033), which suggests that the reduction in ICA blood flow is independently associated with cognitive function. Given the retrospective, cross-sectional design, these findings indicate an association rather than a causal relationship.

Anatomically, the ICA branches intracranially into the anterior cerebral artery (ACA) and the middle cerebral artery (MCA), forming the anterior circulation, and supplies approximately 70% of the total cerebral blood flow (8, 20). The MCA supplies blood to the lateral surface of the frontal lobe, parietal lobe, and upper part of the temporal lobe, which includes the frontoparietal network responsible for attention and working memory, as well as Broca’s and Wernicke’s areas, which are responsible for language function. The ACA supplies blood to the medial surfaces of the frontal and parietal lobes, including the medial surface of the prefrontal cortex and the supplementary motor area, which are involved in decision-making and executive function (21). In other words, the ICA corresponds to the parent artery that supplies blood to most cerebral cortical regions responsible for higher cognitive functions. While transcranial Doppler, which is the modality predominantly used in previous studies, indirectly reflects only the blood flow velocity of the MCA (7, 19), ICA blood flow can comprehensively reflect the supply regions of both the ACA and MCA, and has the advantage of being quantifiable as volumetric flow through carotid duplex ultrasonography. We did not, however, find evidence for domain-specific vulnerability: of the 13 subdomain comparisons, only language and visuospatial construction remained significant after correction for multiple comparisons. This also differs from the domains most consistently affected in a recent meta-analysis, namely attention and executive function (14). The K-MMSE and MoCA-K are brief screening instruments that are not designed to characterize individual cognitive domains, and the present sample provides limited power for 13 simultaneous comparisons; inferences linking these findings to specific neuroanatomical networks would therefore be speculative. As such, cervical SCI may be associated with lower cognitive performance not only through the physical problem of tetraplegia, but also through cerebral perfusion instability caused by autonomic nervous system dysfunction.

In the present study, the presence or absence of orthostatic hypotension diagnosis itself did not show any significant association with cognitive function scores, whereas the change in ICA blood flow was significantly associated with cognitive function. This null finding is compatible with the broader literature: in a meta-analysis of older adults, 14 of 32 studies found no association between orthostatic hypotension and cognition, and the pooled difference in MMSE score was small (22). While orthostatic hypotension is defined based on a decrease in blood pressure upon postural change, ICA blood flow reflects the volume of blood supplied to the brain (8). In patients with SCI, systemic blood pressure and cerebral perfusion may not necessarily correspond because of autonomic nervous system dysfunction and impaired cerebral autoregulation (6, 18). Several alternative explanations must nonetheless be considered. First, the study was not powered to compare the discriminative performance of two markers, and the absence of an association with orthostatic hypotension is not evidence of its absence. Second, the diagnosis of orthostatic hypotension and the ultrasound measurements were made at separate tilt sessions, and orthostatic responses vary between occasions—particularly as management of orthostatic hypotension was initiated after the diagnostic tilt—whereas ΔICA was derived from three averaged measurements at each position within a single session. Third, patients reporting presyncopal symptoms—recorded at the diagnostic tilt, close in time to cognitive assessment and before treatment was initiated—scored lower on the K-MMSE with an effect of the same magnitude as that observed for ΔICA, and in the exploratory joint model symptom status retained statistical significance whereas ΔICA did not. This accords with a previous report that orthostatic hypotension symptom burden was associated with cognitive impairment in chronic SCI (23). Because presyncopal symptoms are plausibly a downstream manifestation of the same postural reduction in cerebral perfusion that ΔICA quantifies, this joint model should not be read as ranking the two markers. We therefore cannot conclude that ICA blood flow is a more sensitive marker than orthostatic hypotension. A more cautious reading is that measures reflecting the physiological consequences of postural change—whether hemodynamic or symptomatic—are associated with cognitive performance, whereas a blood-pressure threshold alone was not in this cohort; bedside symptom inquiry may capture much of the same information, and whether an objective hemodynamic measure adds value where symptom reporting is limited requires prospective evaluation.

ICA blood flow should not be regarded as a direct measure of cerebral perfusion. It is an indirect surrogate that quantifies inflow to the anterior circulation without accounting for intracranial redistribution, collateral supply through the posterior circulation or the circle of Willis, or regional variation in tissue perfusion, and volumetric flow is additionally influenced by arterial carbon dioxide tension, hematocrit, cardiac output, and heart rate, none of which were controlled during tilt testing in this study. Hemoglobin and hematocrit were measured a median of 2 days from ultrasonography and did not differ between groups, but were not measured concurrently; carotid plaque and stenosis were assessed during the routine examination but were not analyzed here, and their potential influence on flow measurement was not evaluated. The observed pattern of change nonetheless offers some mechanistic insight: peak systolic velocity and vessel diameter were unchanged during head-up tilt, whereas end-diastolic and time-averaged mean velocities fell significantly and bilaterally, so the reduction in volume flow was attributable to a fall in diastolic and mean flow velocity rather than to a change in vessel caliber—a pattern consistent with reduced perfusion pressure rather than active vasoconstriction of the extracranial ICA.

Head-up tilt testing is already performed routinely in the early rehabilitation of patients with cervical SCI to identify orthostatic hypotension. In the present cohort the diagnostic tilt test and carotid ultrasonography were performed at separate sessions; combining them into a single examination would require no additional patient visit and would yield a blood-pressure–based diagnosis, a structured record of presyncopal symptoms, and a quantitative measure of the postural change in anterior circulation blood flow. The most plausible near-term application is triage rather than diagnosis. Formal cognitive assessment is not universally performed during acute inpatient rehabilitation, and ΔICA showed moderate discrimination for K-MMSE scores below 24 (AUC 0.718; sensitivity 0.79 and specificity 0.50 at the 21% threshold), a combination of reasonable sensitivity with low specificity that is characteristic of a screening rather than a confirmatory test and would be consistent with using ΔICA to prioritize patients for formal cognitive evaluation. This application remains hypothetical: discrimination was not statistically significant for the MoCA-K (AUC 0.627), the threshold was adopted from a previous study rather than derived independently, and no intervention was tested. Whether identifying patients with large postural reductions in ICA blood flow leads to earlier cognitive assessment, and whether earlier assessment improves rehabilitation outcomes, requires prospective evaluation.

This study has several limitations. First, the sample of 41 patients limits statistical power and precludes adjustment for the full range of potential confounders; covariates in the primary model were therefore restricted to age, sex, and education, with additional variables examined individually in sensitivity analyses. Leave-one-out analysis showed that although the direction of the association was invariant, its statistical significance was sensitive to individual observations. In addition, the cohort was restricted to patients who completed all three assessments; a small number of patients who declined carotid ultrasonography or were too unstable to undergo tilt testing were not captured, their exact number could not be ascertained retrospectively, and some degree of selection cannot be excluded. Second, the definition of ΔICA—the maximum reduction across both sides and both time points—differs from that of the study from which the 21% threshold was adopted, which used the 5-min measurement alone. Unadjusted associations were consistently negative and significant across six alternative definitions, but statistical significance after adjustment varied by definition, and the primary model should be interpreted with this in mind. Third, the interval between cognitive assessment and ultrasonography varied (median 7 days, range −1 to 27), physiological state may have changed within this interval, and treatment of orthostatic hypotension initiated after the diagnostic tilt may have modified the hemodynamic response observed at the ultrasound session; the latter is partly addressed in the medication sensitivity analyses (Supplementary Tables S4, S5). Fourth, depression and fatigue are known to affect performance on cognitive screening instruments but are not routinely screened in this setting and could not be retrieved retrospectively, and pain and sleep disturbance were documented in progress notes but not with standardized instruments and were not analyzed in order to avoid ascertainment bias inherent to retrospective chart abstraction. In addition, although educational attainment was adjusted for, equivalence between groups was supported only within a margin of ±3 years and not ±2 years, and residual confounding by education cannot be excluded. Fifth, the cross-sectional design permits no inference about temporal sequence or causality, and the possibility that both reduced cerebral perfusion and lower cognitive performance reflect a common underlying severity of autonomic dysfunction cannot be excluded. To address these limitations in the future, it will be necessary to conduct a more precise analysis of the region-specificity of cerebral blood flow changes by combining imaging studies such as Brain SPECT or fMRI.

Lower cognitive performance and psychiatric symptoms such as depression, anxiety, and post-traumatic stress disorder are commonly observed among patients with cervical SCI, and can compound these difficulties and reduce participation in rehabilitation treatment (24–26). Therefore, future studies are needed to confirm the association with cognitive function through a comprehensive evaluation including psychological variables such as depression and anxiety.

## 5 Conclusion

Overall, the present study demonstrated that a decrease in ICA blood flow was significantly associated with lower cognitive performance in patients with cervical SCI. Patients with a decrease in ICA blood flow of 21% or more had significantly lower cognitive assessment scores than those without such a decrease. Given the retrospective, cross-sectional design, these findings should be interpreted as associative. These results support the potential value of conducting assessments of ICA blood flow and cognitive function tests early in the rehabilitation process of patients with cervical SCI, most plausibly to identify patients who should be prioritized for formal cognitive assessment; this application requires prospective evaluation.

## Data Availability

The raw data supporting the conclusions of this article will be made available by the authors, without undue reservation.
