# Supplementary material for: Relationship between orthostatic hypotension, internal carotid artery blood flow, and cognition in patients with cervical spinal cord injury
Source: Front Neurol. 2026 Sep 14;17:1915120. doi: 10.3389/fneur.2026.1915120 (PMC13616682; doi:10.3389/fneur.2026.1915120)
Supplement: Supplementary file 1 [file Data_Sheet_1.DOCX]

**Supplementary Material**

**Supplementary Table S1.** Sensitivity of the adjusted association between dichotomized ΔICA and cognitive scores to the choice of cutoff

| **Cutoff (%)** | **n (≥ / <)** | **K-MMSE β** | **P** | **MoCA-K β** | **P** |
| --- | --- | --- | --- | --- | --- |
| 10 | 33 / 8 | −2.44 | 0.105 | −2.74 | 0.214 |
| 15 | 30 / 11 | −3.47 | 0.008 | −3.90 | 0.045 |
| 20 | 26 / 15 | −2.61 | 0.027 | −3.90 | 0.023 |
| 21 (primary) | 26 / 15 | −2.61 | 0.027 | −3.90 | 0.023 |
| 25 | 18 / 23 | −2.36 | 0.046 | −3.97 | 0.019 |
| 30 | 14 / 27 | −1.48 | 0.265 | −3.32 | 0.080 |
| 35 | 10 / 31 | −1.18 | 0.389 | −2.06 | 0.298 |

β is the adjusted difference in score between patients at or above and below each cutoff, from linear regression adjusted for age, sex, and years of education. The association remained significant between the 15% and 25% cutoffs for both instruments and was attenuated at the extremes.

**Supplementary Table S2.** Association between ΔICA and cognitive scores under six alternative definitions of ΔICA

| **Definition of ΔICA** | **r** | **P** | **Adj. P** | **r** | **P** | **Adj. P** |
| --- | --- | --- | --- | --- | --- | --- |
| Maximum reduction across both sides and both time points (primary) | −0.445 | 0.004 | 0.038 | −0.435 | 0.004 | 0.033 |
| 5-minute measurement, maximum of the two sides | −0.378 | 0.015 | 0.239 | −0.423 | 0.006 | 0.085 |
| 1-minute measurement, maximum of the two sides | −0.440 | 0.004 | 0.055 | −0.346 | 0.027 | 0.217 |
| 5-minute measurement, summed bilateral flow | −0.391 | 0.012 | 0.128 | −0.365 | 0.019 | 0.154 |
| 5-minute measurement, mean of the two sides | −0.357 | 0.022 | 0.172 | −0.345 | 0.027 | 0.155 |
| Maximum across time points, summed bilateral flow | −0.398 | 0.010 | 0.103 | −0.375 | 0.016 | 0.120 |

The first three data columns refer to the K-MMSE and the last three to the MoCA-K. r, Pearson correlation coefficient; Adj. P, P value for ΔICA modeled continuously (per 10-percentage-point reduction) in linear regression adjusted for age, sex, and years of education. Unadjusted correlations were negative and statistically significant under all six definitions for both instruments (r = −0.345 to −0.445); statistical significance after adjustment varied by definition.

**Supplementary Table S3.** Diagnostics of the primary regression models and leave-one-out analysis

**(a) Variance inflation factors (adjusted model)**

| **Variable** | **VIF** |
| --- | --- |
| ΔICA (per 10 percentage points) | 1.12 |
| Age | 1.48 |
| Sex | 1.31 |
| Years of education | 1.98 |

**(b) Model diagnostics and leave-one-out analysis**

|  | **K-MMSE model** | **MoCA-K model** |
| --- | --- | --- |
| Breusch–Pagan test (F statistic), P | 0.926 | 0.186 |
| Maximum Cook’s distance | 0.359 | 0.157 |
| Shapiro–Wilk test of residuals, P | 0.280 | 0.005 |
| Leave-one-out: iterations with unchanged coefficient direction | 41 / 41 | 41 / 41 |
| Leave-one-out: iterations with P > 0.05 | 11 / 41 | 5 / 41 |
| Leave-one-out: range of ΔICA coefficient | −1.14 to −0.62 | −1.49 to −0.87 |

Models include ΔICA (per 10 percentage points), age, sex, and years of education. Because the residuals of the MoCA-K model deviated from normality, bootstrap confidence intervals (5,000 resamples, percentile method) are reported alongside parametric intervals in Table 5. In leave-one-out analyses, each of the 41 patients was omitted in turn and the model refitted.

**Supplementary Table S4.** Sensitivity models: individual adjustment for non-medication covariates

| **Covariate added to the primary model** | **K-MMSE β** | **P** | **MoCA-K β** | **P** |
| --- | --- | --- | --- | --- |
| Time since injury | −1.03 | 0.009 | −1.31 | 0.029 |
| Orthostatic hypotension (diagnosis) | −0.90 | 0.027 | −1.24 | 0.037 |
| Hemoglobin | −0.81 | 0.039 | −1.19 | 0.028 |
| Injury completeness (AIS A/B versus C/D) | −0.86 | 0.035 | −1.28 | 0.031 |

Each covariate was added individually to the primary model (ΔICA per 10 percentage points, age, sex, and years of education). β and P refer to the ΔICA coefficient in each model.

**Supplementary Table S5.** Sensitivity analyses for medication variables

| **Analysis** | **K-MMSE β** | **P** | **MoCA-K β** | **P** |
| --- | --- | --- | --- | --- |
| Adjustment for anticholinergic burden (ACB score) | −0.83 | 0.039 | −1.23 | 0.036 |
| Adjustment for sedative medication | −0.84 | 0.040 | −1.23 | 0.039 |
| Adjustment for gabapentinoid use | −0.78 | 0.050 | −1.20 | 0.041 |
| Adjustment for antihypertensive medication | −0.82 | 0.041 | −1.22 | 0.035 |
| Adjustment for midodrine or fludrocortisone use | −0.93 | 0.030 | −1.15 | 0.062 |
| Adjustment for total number of medications | −0.73 | 0.080 | −0.94 | 0.110 |
| Exclusion of patients on midodrine or fludrocortisone (n = 26) | −1.10 | 0.057 | −0.88 | 0.232 |

In adjustment analyses, each medication variable was added individually to the primary model; in the exclusion analysis, the primary model was refitted after excluding the 15 patients receiving midodrine or fludrocortisone at the time of ultrasonography. β and P refer to the ΔICA coefficient in each analysis. Anticholinergic exposure was modeled as the Anticholinergic Cognitive Burden (ACB) score. Cognitive variables were assessed on the day of cognitive testing and hemodynamic variables on the day of ultrasonography. Systemic corticosteroids (five patients, all in Group A, prescribed for cutaneous adverse drug reactions) could not be separated from the group effect owing to complete separation; with corticosteroid use added, the ΔICA coefficient was −0.57 (P = 0.143) for the K-MMSE and −0.88 (P = 0.121) for the MoCA-K, and corticosteroid use may act as a marker of acute-phase severity.

**Supplementary Figure S1.**

**Supplementary Figure S1.** Receiver operating characteristic curves for ΔICA against four outcomes: K-MMSE below 24, MoCA-K below 23, presyncopal symptoms, and the diagnosis of orthostatic hypotension. Circles mark the a priori 21% threshold and triangles the Youden-derived optimum for each outcome. ΔICA discriminated K-MMSE scores below 24 (area under the curve 0.718, bootstrap 95% CI 0.545–0.878; Youden-optimal threshold 22.8%); discrimination was not statistically significant for the other three outcomes (MoCA-K, 0.627, 0.442–0.813; presyncopal symptoms, 0.656, 0.476–0.821; orthostatic hypotension, 0.609, 0.410–0.797).
